# Medical Cannabis Use Reduces Opioid Prescriptions in Patients With Osteoarthritis

**DOI:** 10.7759/cureus.21564

**Published:** 2022-01-24

**Authors:** Bryan Renslo, Ari Greis, Conan S Liu, Anjithaa Radakrishnan, Asif M Ilyas

**Affiliations:** 1 Department of Medical Cannabis, Sidney Kimmel Medical College at Thomas Jefferson University, Philadelphia, USA; 2 Rothman Orthopaedic Institute Foundation for Opioid Research & Education, Rothman Orthopaedic Institute, Philadelphia, USA

**Keywords:** prescription opioid, opioid use, cannabis use, cannabis (marijuana), osteoarthritis (oa), chronic pain management

## Abstract

Background

Osteoarthritis (OA) can result in significant pain, requiring pain management with opioids. Medical cannabis (MC) has the potential to be an alternative to opioids for chronic pain conditions. This study investigates whether MC used in the management of OA-related chronic pain can reduce opioid utilization.

Methods

Forty patients with chronic OA pain were certified for MC. Average morphine milligram equivalents (MME) per day of opioid prescriptions filled within the six months prior to MC certification was compared to that of the six months after. Visual analog scale (VAS) for pain and Global Health scores were measured at baseline, three, and six months post MC certification.

Results

Average MME/day decreased from 18.2 to 9.8 (n=40, p<0.05). The percentage of patients who dropped to 0 MME/day was 37.5%. VAS scores decreased significantly at three and six months, and Global Physical Health score increased significantly by three months.

Conclusions

MC reduces opioid prescription for patients with chronic OA pain and improves pain and quality of life.

## Introduction

The United States (US) currently faces an opioid crisis with opioid-related deaths nearly quadrupling between 1999 and 2015 and 37.8% of adults using opioids in 2015 [1]. Opioids have shown statistically significant but small improvements in treating chronic pain at the cost of dose-dependent risks of substance abuse disorders, addiction, overdose, and death [2]. Opioids are heavily prescribed for orthopaedic indications and often for the surgical and non-surgical management of osteoarthritis (OA) [3]. In fact, the prescription of opioids for OA increased from 13.4% to 17% between 2007 to 2014, despite increased awareness of their adverse effects [3]. Furthermore, most guidelines continue to recommend opioids for advanced OA pain [4].

Medical cannabis (MC) is a new therapeutic option for chronic pain. As of 2020, it has been approved for the treatment of pain in over 30 states across the US [5]. MC has been shown to treat chronic noncancer pain, neuropathic pain, and multiple sclerosis-related spasticities, and is generally well-tolerated and safe to use [6-9]. MC has also been shown to be effective in treating orthopaedic pain when compared to a placebo; however, studies have yet to show efficacy when compared to an active comparator [10]. There are limited studies into the use of MC for the management of chronic pain caused by musculoskeletal etiologies such as OA, in part due to hesitancy from physicians to prescribe it [11]. Preliminary studies in patients with OA and rheumatoid arthritis have shown that the cannabinoid receptor system is present in the synovium of affected joints, providing evidence that MC has potential for therapeutic pain management in these patients [12].

Population studies show that MC legalization has been associated with reduced mortality due to opioid overdose, reduced opioid-related hospitalizations, and decreased opioid prescription [13]. However, there is so far insufficient evidence to show that MC can be an effective replacement for opioids. Therefore, this study focused on the association between MC use and opioid utilization for the management of chronic pain due to OA. The study hypothesis was that MC certification and use for chronic pain from OA would result in a decrease of opioid prescriptions filled.

## Materials and methods

Patient outcome measures for this study were obtained by querying the Rothman Orthopaedic Cannabis Data Repository (ROCDR). The establishment of the data repository was approved by the Institutional Review Board of Thomas Jefferson University, Philadelphia, Pennsylvania (protocol #19D.159). Patients with non-spinal chronic OA diagnoses who were certified for MC at the Medical Cannabis Department of the Rothman Orthopaedic Institute between February 2018 and July 2019 were prospectively enrolled and included. Inclusion criteria for study analysis included patients actively consuming opioids for chronic pain, and OA was the primary contributor to their pain. Exclusion criteria included patients having undergone surgery within six months before or after MC certification, and not consuming opioids at the time of MC certification. Patient demographic information was collected. Outcome measures were collected at baseline and at three and six months post-MC using the Patient-Reported Outcomes Measurement Information System (PROMIS) questionnaire and included visual analog scale (VAS) pain score, Global Mental Health (GMH) quality of life (QoL) score, and Global Physical Health (GPH) QoL score. Due to variability in the site of joint pain, global pain outcome measures were used instead of OA joint-specific measures. Data on the route of MC administration and adverse effects were retrospectively collected during the three and six-month follow-up visits and analyzed.

All physicians who were involved with MC certification had previously undergone a four-hour Continuing Medical Education (CME) course and applied to the Pennsylvania Department of Health in order to become approved practitioners as mandated by the state of Pennsylvania [14]. MC was certified for patients who met the criteria by being a resident of Pennsylvania and suffering from one of the 23 state-approved qualifying medical conditions [14]. During the certification visit, the chemical constituents of MC, routes of delivery, and optimal dosing parameters were reviewed with the patient. Patients were required to sign an informed consent form and the potential risks of using MC were reviewed. For patients naïve to cannabis, it was recommended they start with low dosages of tetrahydrocannabinol (THC), oftentimes combined with cannabidiol (CBD). An oral route of delivery, often with a sublingual tincture, and/or topical cannabinoids were recommended over vaporization, and smoking was not endorsed. Once certified, patients could purchase an MC identification card from the Pennsylvania Department of Health and shop at state-approved cannabis dispensaries.

Data regarding filled prescriptions of controlled substances were gathered using Pennsylvania’s Prescription Drug Monitoring Program (PDMP) system. PDMP is a state-run program that collects information on all filled prescriptions for controlled substances and can be accessed online by licensed providers. Study data were logged into a password-protected Microsoft Excel (Microsoft Corp., Redmond, Washinton) database.

Data on opioid prescriptions that were filled within six months before and after MC certification for all enrolled patients were collected. Opioid prescriptions filled were calculated based on the average morphine milligram equivalents (MME) filled per day over the six-month period before and after MC certification. Total MME per patient was calculated by multiplying PDMP-provided raw daily MME by the number of days the patient had an active opioid prescription. Daily opioid consumption was normalized by dividing each patient’s total MME prescribed in a six-month period by the total number of days within six months. The six-month period was chosen to normalize opioid prescription variability for their chronic pain over a longer time period of time. Primary study outcome measures consisted of the change in opioid prescriptions filled pre- and post-MC certification and use. Secondary study outcome measures included pain and QoL scores.

Statistics were calculated within Microsoft Excel using a paired, two-tailed T-test for paired data and a two-tailed T-test with unequal variance for non-paired data. An alpha threshold of alpha = 0.05 was applied for all unique significance tests.

## Results

**Figure 1:**
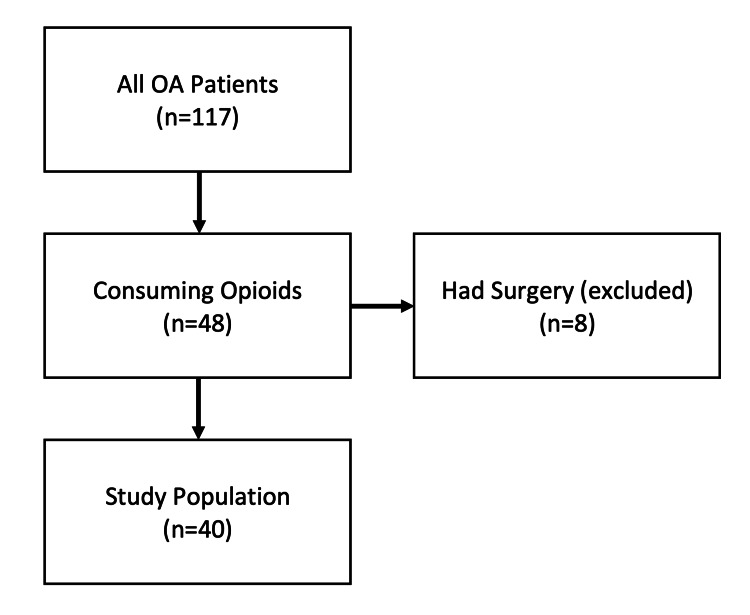
Patients who filled controlled substance prescriptions within six months before medical cannabis certification Of all patients with osteoarthritis (n=117), 48 were actively consuming opioids. Patients who had surgery within six months of medical cannabis certification (n=8) were excluded. Our final study population included 40 patients.

A total of 632 patients were certified for MC for a musculoskeletal pain diagnosis between February 2018 and July 2019. Of those patients, 117 patients had a diagnosis related to OA, with 48 having filled an opioid prescription within the six months prior to MC certification (Figure 1). There were eight patients who were then removed due to surgery within the time frame of the study. Among the 40 patients finally included in the study, 31 (77.5%) were female and nine (22.5%) were male. The average age at the date of MC certification was 67.9 (46-90) years. Primary sites of pain for each patient are listed in Table 1. One-way ANOVA revealed no statistically significant difference in baseline MME/day (F(3,36)=0.46, p=0.71), VAS pain score (F(3,32)=1.35, p=0.27), GMH score (F(3,32)=1.04, p=0.39), or GPH score (F(3,32)=2.58, p=0.07) between patients with different sites of pain.

**Table 1:** Primary site of pain

Category	N
Knee	18
Shoulder	12
Hip	9
Hand	1

**Figure 2:**
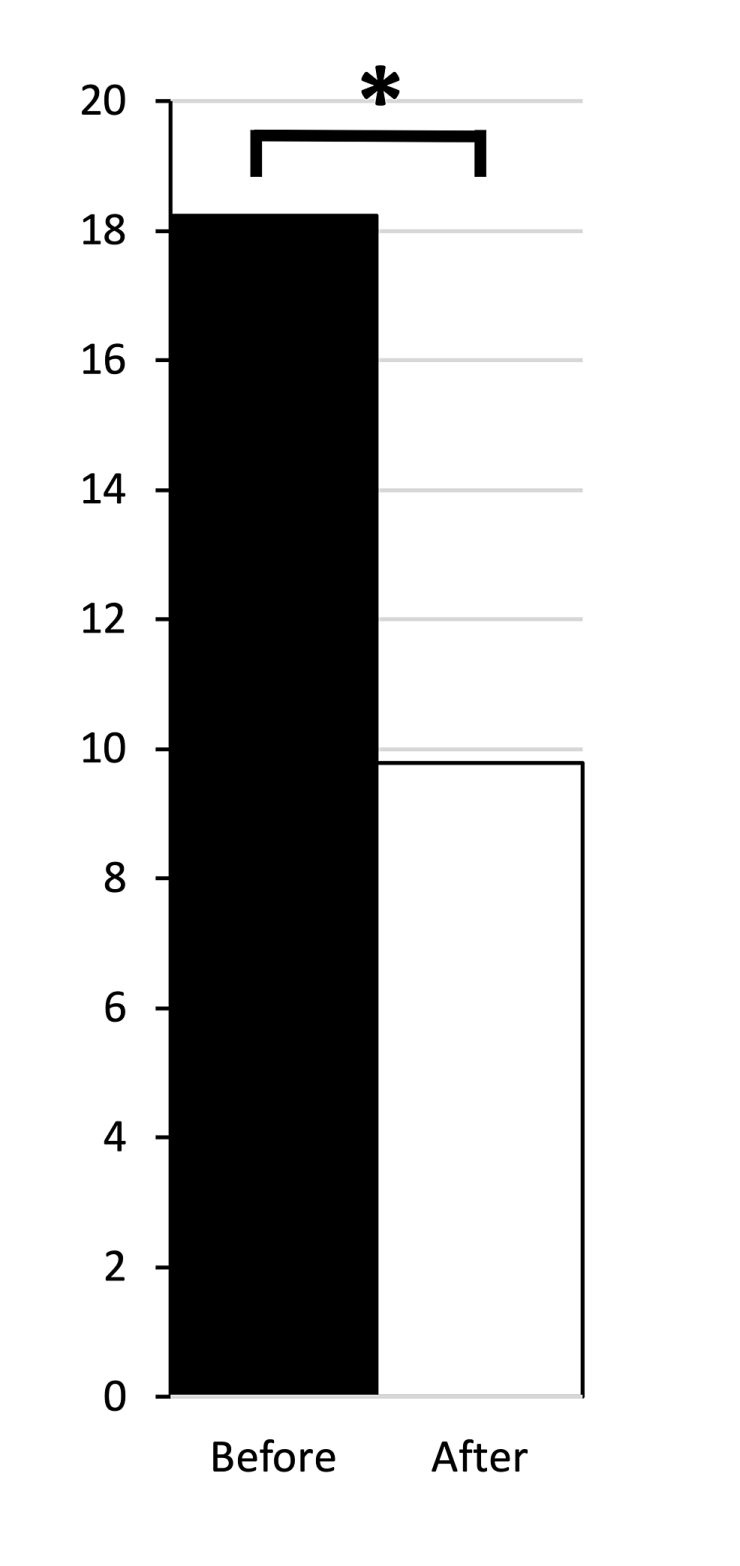
Opioid group shows decreased opioid prescriptions filled post medical cannabis certification All patients on opioids showed a drop in morphine milligram equivalents per day (MME/day) from 18.2 to 9.8 (n=40, *p<0.05).

For all patients, average MME/day dropped from 18.2 to 9.8 (n=40, p<0.05) between six months pre-MC to six months post-MC use (Figure 2). Moreover, the percentage of those patients who dropped to 0 MME/day following MC certification was 37.5%. The average drop in MME/day was 46.3%.

**Figure 3:**
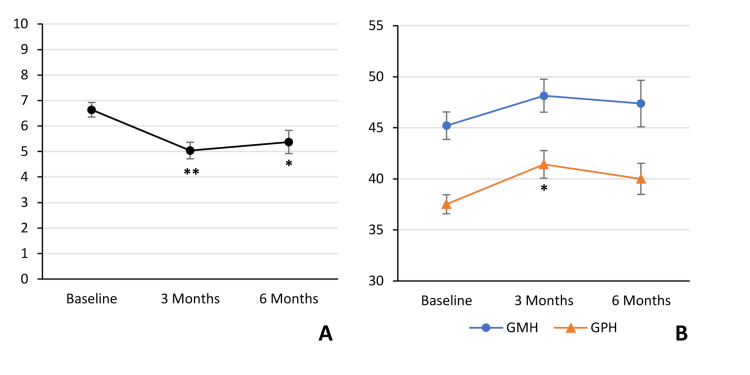
Pain and Quality of Life scores are improved following medical cannabis certification Visual Analog Scale (VAS) pain score, Global Mental Health (GMH), and Global Physical Health (GPH) were measured at baseline (n=36) and at three months (n=26), and six months (n=16) following MC certification. VAS pain score decreased significantly from 6.6 at baseline to 5.0 (**p<0.01) at three months and 5.4 (*p<0.05) at six months (A). GMH increased insignificantly from 45.2 to 48.1 (p=0.17) at three months and 47.4 (p=0.42) at six months (B). GPH increased significantly from 37.5 to 41.4 (*p<0.05) at three months and increased insignificantly to 40.0 (p=0.18) at six months (B). MC: medical cannabis

VAS pain score and QoL scores were measured at baseline and at three and six months (n=16) post MC certification. Compared to baseline (prior to the initiation of MC therapy), VAS pain score decreased significantly from 6.6 to 5.0 (p<0.01) and 5.4 (p<0.05) at three and six months, respectively (Figure 3A). GMH score increased insignificantly from 45.2 to 48.1 (p=0.17) and 47.4 (p=0.42) at three and six months, respectively (Figure 3B). GPH score increased significantly from 37.5 to 41.4 (p<0.05) at three months and increased insignificantly to 40.0 (p=0.18) at six months (Figure 3B).

**Figure 4:**
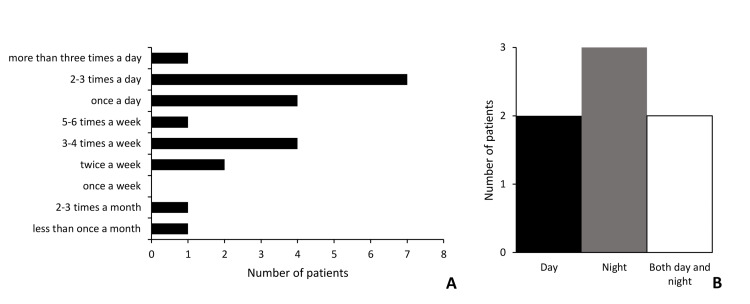
Patterns of medical cannabis use Patients were queried regarding patterns of MC use (n=21). When asked how often they take MC, 33.3% (n=7) said they take MC two to three times per day, followed by 19.0% (n=4) once a day, and 19.0% (n=4) three to four times a week (A). Among respondents who take multiple routes of MC (n=7), two patients take MC only during the day, three take MC only at night, and two take MC during the day and at night (B). MC: medical cannabis

Various routes of MC administration were used including vaporized oil, vaporized flower, and oral, topical, and sublingual tincture. We collected data on the route of MC administration for 33 (82.5%) patients. Of these, 21 (63.6%) used only a single route, 11 (33.3%) used two routes, one (3%) used three routes. The most commonly used route of administration was sublingual tincture (n=22, 66.7%) followed by topical (n=11, 33.3%), vaporized oil (n=7, 21.2%), oral (n=3, 9.1%), and vaporized flower (n=3, 9.1%) (Table 2). Among respondents using only a single route of administration, sublingual tincture was used most commonly, with 15 patients (71.4%) using it (Table 3). Regarding patterns of MC use (n=21) among respondents, 33.3% (n=7) said they use MC two to three times per day, which was most common (Figure 4A), 19.0% (n=4) said they use MC once a day, and the same number of patients 19.0% (n=4) said they use MC three to four times per week. Among respondents who use multiple routes of MC (n=7), the majority (n=5) said they use MC either during the day or at night only, suggesting they use both routes simultaneously (Figure 4B).

**Table 2:** Medical cannabis routes of administration

Route of Administration	N	%
Sublingual Tincture	22	66.7%
Topical	11	33.3%
Vaporized Oil	7	21.2%
Oral	3	9.1%
Vaporized Flower	3	9.1%

**Table 3:** Medical cannabis routes of administration for single-route users

Route of Administration	N	%
Sublingual Tincture	15	71.4%
Vaporized Oil	2	9.5%
Topical	2	9.5%
Oral	2	9.5%
Vaporized Flower	0	0.0%

Data on adverse effects were collected for 21 (52.5%) patients. Of the respondents, 12 patients (57.1%) did not feel intoxicated or high, and nine patients (42.9%) felt intoxicated or high. Of the patients who felt intoxicated or high, three (1.4%) said that it did not interfere with their daily activities, three (1.4%) said that it made their day even better, and three (1.4%) said that they did not like it or that it interfered with their daily activities (Table 4).

**Table 4:** Patient-reported adverse effects of medical cannabis

Category	N	%
All Respondents	21	100
I don’t feel intoxicated/high	12	57.1
I feel intoxicated/high	9	42.9
It doesn’t interfere with my daily activities	3	1.4
It makes my day even better (I enjoy it)	3	1.4
I don’t like it (or it interferes with my daily activities)	3	1.4

## Discussion

The study hypothesis was upheld as patients certified for MC for the management of their chronic OA pain filled statistically less opioid prescriptions six months after certification than the six months before it. In addition, over a third of the patients stopped filling opioid prescriptions entirely. Moreover, VAS pain scores improved at three and six months post MC while GPH improved at three months post MC. Our findings indicate that providing access to MC helps patients with chronic pain due to OA reduce their levels of opioid usage in addition to improving pain and QoL. Furthermore, a majority of patients did not feel intoxicated or high from MC, and of those who did, only a small percentage said it interfered with their daily activities. It should be noted that our findings on adverse effects are limited given the variability in patient MC consumption.

A 28.2% change in MME/day has been suggested as a minimal clinical important difference (MCID) [15]. Based on our data, MME/day dropped at a level that would meet the criteria for MCID. MCID in pain on the VAS is reported as 13 out of 100 [16]. VAS pain decreased by a level that would be considered clinically significant at three months but not six months. MCID in GMH and GPH scores has not been described for OA [17].

Our findings support the literature in that MC reduces the use of opioids for the treatment of chronic pain [10,18-24]. The literature regarding MC for chronic pain for orthopaedic conditions is currently unclear [12,25]. Most studies utilize patient surveys, which are inherently subjective, to measure opioid use [18-22,26]. Additionally, very few studies investigate how the use of MC affects opioid usage specifically for chronic pain due to OA [11,26,27]. Our study provides an objective quantification metric of opioid utilization and adds to current literature regarding MC use for chronic pain due to OA.

One study looking at a cohort of patients undergoing primary joint arthroplasty showed that tetrahydrocannabinol (THC) and other cannabidiol (CBD) product use was not associated with differences in length of opioid use, total MME used, average postoperative pain score, percentage of patients requiring a refill of narcotics, or length of stay in the perioperative period [26]. This study differs from our own in that it focused on the perioperative period when levels of pain are less consistent. Furthermore, this study did not examine the individual patient responses to MC use.

Opioids are still routinely used for OA conditions despite issues of tolerability, dependency, and abuse [3,4]. MC is currently recommended as an adjunct for first and second-line medications for chronic musculoskeletal pain should those options fail [28]. Based on our findings, the introduction of MC for patients with low levels of opioid utilization has a high chance of decreasing opioid utilization and even eliminating the need for opioid medications to control their pain altogether. Our findings suggest that MC should be considered for patients with chronic pain due to OA in order to decrease opioid use. Moreover, because MC has a superior safety profile and minimizes the risk of potentially fatal overdose, MC can also be considered a viable option prior to initiating opioid prescriptions.

Physicians are often hesitant to prescribe MC due to uncertainty of its efficacy and limited experience with its use. In a study conducted in Germany, only 1.9% of patients with nociceptive, neuropathic, or neuroplastic pain were prescribed MC despite its beneficial outcomes [29]. There is difficulty in developing solid guidelines for MC usage due to variability in the literature regarding efficacy, route, dosing, and safety. The fact that MC continues to be a Schedule 1 drug limits physicians from prescribing MC formally and conducting controlled trials. Furthermore, the current climate of MC prescription allows unlicensed healthcare proxies to provide advice for MC use in lieu of licensed physicians [30]. Patients who are certified for MC as part of state-approved MC programs are merely granted access to MC and thus have the ability to choose their own dosage, frequency, and route of delivery for their medication. This study begins to elucidate specific indications for MC including chronic pain due to OA, and it also looks into which opioid usage profiles would be most responsive to MC. However, future guidelines regarding MC cannot be established without further study into MC indications, dosing, route of administration, and long-term efficacy and side effect profiles.

This study has several limitations. It was an uncontrolled observational study. Our population was fairly small and limits generalizability, so larger follow-up is warranted. Tracking filled opioid prescriptions does not necessarily indicate the actual use of opioids and only serves as a proxy for opioid consumption. Furthermore, prescriptions are often shared, and we could not account for the acquisition and use of illicit opioids. The dosage and route of MC use were not tracked and are difficult to ascertain, as it is typically up to the patient once access is granted. Our study only follows patients for six months post MC certification and may miss attrition of MC effectiveness. Data on MC toxicity and side effects are difficult to generalize given the variability in patient MC consumption.

## Conclusions

Patients with chronic pain due to OA who were certified for MC filled significantly less opioid prescriptions post MC certification compared to pre-MC certification. Over one-third of patients stopped filling opioid prescriptions altogether. Pain and QoL measures were improved following MC certification. Sublingual tincture was the most commonly used route of MC administration.

## References

[1] Han B, Compton WM, Blanco C, Crane E, Lee J, Jones CM (2017). Prescription opioid use, misuse, and use disorders in U.S. adults: 2015 National Survey on Drug Use and Health. Ann Intern Med.

[2] Busse JW, Wang L, Kamaleldin M (2018). Opioids for chronic noncancer pain: a systematic review and meta-analysis. JAMA.

[3] DeMik DE, Bedard NA, Dowdle SB, Burnett RA, McHugh MA, Callaghan JJ (2017). Are we still prescribing opioids for osteoarthritis?. J Arthroplasty.

[4] Fitzcharles MA, Baerwald C, Ablin J, Häuser W (2016). Efficacy, tolerability and safety of cannabinoids in chronic pain associated with rheumatic diseases (fibromyalgia syndrome, back pain, osteoarthritis, rheumatoid arthritis): a systematic review of randomized controlled trials. Schmerz.

[5] (2022). NCSL: State medical cannabis laws. https://www.ncsl.org/research/health/state-medical-marijuana-laws.aspx.

[6] Romero-Sandoval EA, Kolano AL, Alvarado-Vázquez PA (2017). Cannabis and cannabinoids for chronic pain. Curr Rheumatol Rep.

[7] Hill KP (2015). Medical marijuana for treatment of chronic pain and other medical and psychiatric problems: a clinical review. JAMA.

[8] Lynch ME, Campbell F (2011). Cannabinoids for treatment of chronic non-cancer pain; a systematic review of randomized trials. Br J Clin Pharmacol.

[9] Lynch ME, Ware MA (2015). Cannabinoids for the treatment of chronic non-cancer pain: an updated systematic review of randomized controlled trials. J Neuroimmune Pharmacol.

[10] Madden K, George A, van der Hoek NJ, Borim FM, Mammen G, Bhandari M (2019). Cannabis for pain in orthopedics: a systematic review focusing on study methodology. Can J Surg.

[11] O'Brien M, McDougall JJ (2018). Cannabis and joints: scientific evidence for the alleviation of osteoarthritis pain by cannabinoids. Curr Opin Pharmacol.

[12] Richardson D, Pearson RG, Kurian N (2008). Characterisation of the cannabinoid receptor system in synovial tissue and fluid in patients with osteoarthritis and rheumatoid arthritis. Arthritis Res Ther.

[13] Powell D, Pacula RL, Jacobson M (2018). Do medical marijuana laws reduce addictions and deaths related to pain killers?. J Health Econ.

[14] (2020). Pennsylvania Department of Health: Pennsylvania medical marijuana program. https://www.health.pa.gov/topics/programs/Medical%20Marijuana/Pages/Medical%20Marijuana.aspx.

[15] Goudman L, Smedt A, Forget P, Moens M (2020). Determining the minimal clinical important difference for medication quantification scale iii and morphine milligram equivalents in patients with failed back surgery syndrome. J Clin Med.

[16] Todd KH, Funk KG, Funk JP, Bonacci R (1996). Clinical significance of reported changes in pain severity. Ann Emerg Med Apr.

[17] Busija L, Ackerman IN, Haas R (2020). Adult measures of general health and health-related quality of life. Arthritis Care Res (Hoboken).

[18] Boehnke KF, Scott JR, Litinas E, Sisley S, Williams DA, Clauw DJ (2020). High-frequency medical cannabis use is associated with worse pain among individuals with chronic pain. J Pain.

[19] Reiman A, Welty M, Solomon P (2017). Cannabis as a substitute for opioid-based pain medication: patient self-report. Cannabis Cannabinoid Res.

[20] Haroutounian S, Ratz Y, Ginosar Y, Furmanov K, Saifi F, Meidan R, Davidson E (2016). The effect of medicinal cannabis on pain and quality-of-life outcomes in chronic pain: a prospective open-label study. Clin J Pain.

[21] Nielsen S, Sabioni P, Trigo JM (2017). Opioid-sparing effect of cannabinoids: a systematic review and meta-analysis. Neuropsychopharmacology.

[22] Lucas P, Walsh Z (2017). Medical cannabis access, use, and substitution for prescription opioids and other substances: a survey of authorized medical cannabis patients. Int J Drug Policy.

[23] Lopez CD, Boddapati V, Jobin CM, Hickernell TR (2021). State medical cannabis laws associated with reduction in opioid prescriptions by orthopaedic surgeons in Medicare Part D Cohort. J Am Acad Orthop Surg.

[24] Takakuwa KM, Hergenrather JY, Shofer FS, Schears RM (2020). The impact of medical cannabis on intermittent and chronic opioid users with back pain: how cannabis diminished prescription opioid usage. Cannabis Cannabinoid Res.

[25] Katz-Talmor D, Katz I, Porat-Katz BS, Shoenfeld Y (2018). Cannabinoids for the treatment of rheumatic diseases - where do we stand?. Nat Rev Rheumatol.

[26] Runner RP, Luu AN, Nassif NA, Scudday TS, Patel JJ, Barnett SL, Gorab RS (2020). Use of tetrahydrocannabinol and cannabidiol products in the perioperative period around primary unilateral total hip and knee arthroplasty. J Arthroplasty.

[27] Yip K, Oettinger J (2020). Why are we still using opioids for osteoarthritis?. Int J Clin Pract.

[28] Johal H, Vannabouathong C, Chang Y, Zhu M, Bhandari M (2020). Medical cannabis for orthopaedic patients with chronic musculoskeletal pain: does evidence support its use?. Ther Adv Musculoskelet Dis.

[29] Bialas P, Drescher B, Gottschling S (2019). Cannabis-based medicines for chronic pain: indications, selection of drugs, effectiveness and safety: Experiences of pain physicians in Saarland [Article in German]. Schmerz.

[30] Corroon J, Sexton M, Bradley R (2019). Indications and administration practices amongst medical cannabis healthcare providers: a cross-sectional survey. BMC Fam Pract.

